# Natural tick-borne encephalitis in 2 Huacaya alpacas (*Vicugna pacos*)

**DOI:** 10.1177/03009858251362432

**Published:** 2025-08-19

**Authors:** Denise Thaller, Claudia Schulz, Angelika Auer, Zoltán Bagó, Sandra Revilla-Fernández, Michael D. Mansfeld, Kaspar Matiasek, Andrea Klang

**Affiliations:** 1 University of Veterinary Medicine Vienna, Vienna, Austria; 2 Institute for Veterinary Disease Control, Mödling, Austria; 3 Carinthian Institute for Food Safety and Veterinary Medicine, Klagenfurt, Austria; 4 Ludwig Maximilian University, Munich, Germany

**Keywords:** arboviruses, camelids—South American, central nervous system, encephalitis—tick-borne, immunohistochemistry, polymerase chain reaction, RNAscope in situ hybridization, sequence analysis

## Abstract

Although rare, tick-borne encephalitis (TBE) is one of the most important and commonly fatal viral diseases affecting the central nervous system (CNS). This arboviral disease is transmitted by ticks and prevalent in widespread parts of Eurasia. Besides humans, several domestic animals such as dogs, horses, and ruminants can also be infected. To our knowledge, there have been no reports of TBE in South American camelids, so far. Here, we present 2 cases of Huacaya alpacas with progressive, therapy-resistant neurologic signs, which were euthanized and submitted for necropsy. Histologic examination of the CNS revealed a moderate lymphohistiocytic meningoencephalomyelitis characterized by perivascular cuffing, glial cell proliferation, neuronal degeneration, and neuronophagia. Tick-borne encephalitis virus (TBEV) infection was confirmed by polymerase chain reaction (PCR), sequencing, immunohistochemistry, and RNAscope in situ hybridization. TBEV should be included as a differential diagnosis in alpacas from endemic regions presenting with neurologic signs.

Generalized neurologic disease is common in alpacas and other South American camelids.^27^ However, viral infections of the central nervous system (CNS) are infrequently reported and comprise rabies,^4,16,25,28^ equine herpesvirus-1,^17^ borna disease virus,^9^ West Nile virus,^8,29^ Eastern equine encephalomyelitis virus,^14^ and louping ill virus.^7,13^ Currently, some of these diseases, such as louping ill and Eastern equine encephalitis, are restricted to certain continents or countries such as the United States of America^14^ and Great Britain.^7,13^ Tick-borne encephalitis virus (TBEV), an endemic arbovirus in the *Flaviviridae* family, is an emerging health problem in people. Primarily transmitted by hard ticks, TBEV has been identified in at least 27 European and Asian countries.^5^ Here, we present 2 cases of tick-borne encephalitis (TBE) in alpacas detected in Austria in 2022.

## Case 1

**Figure 1.**
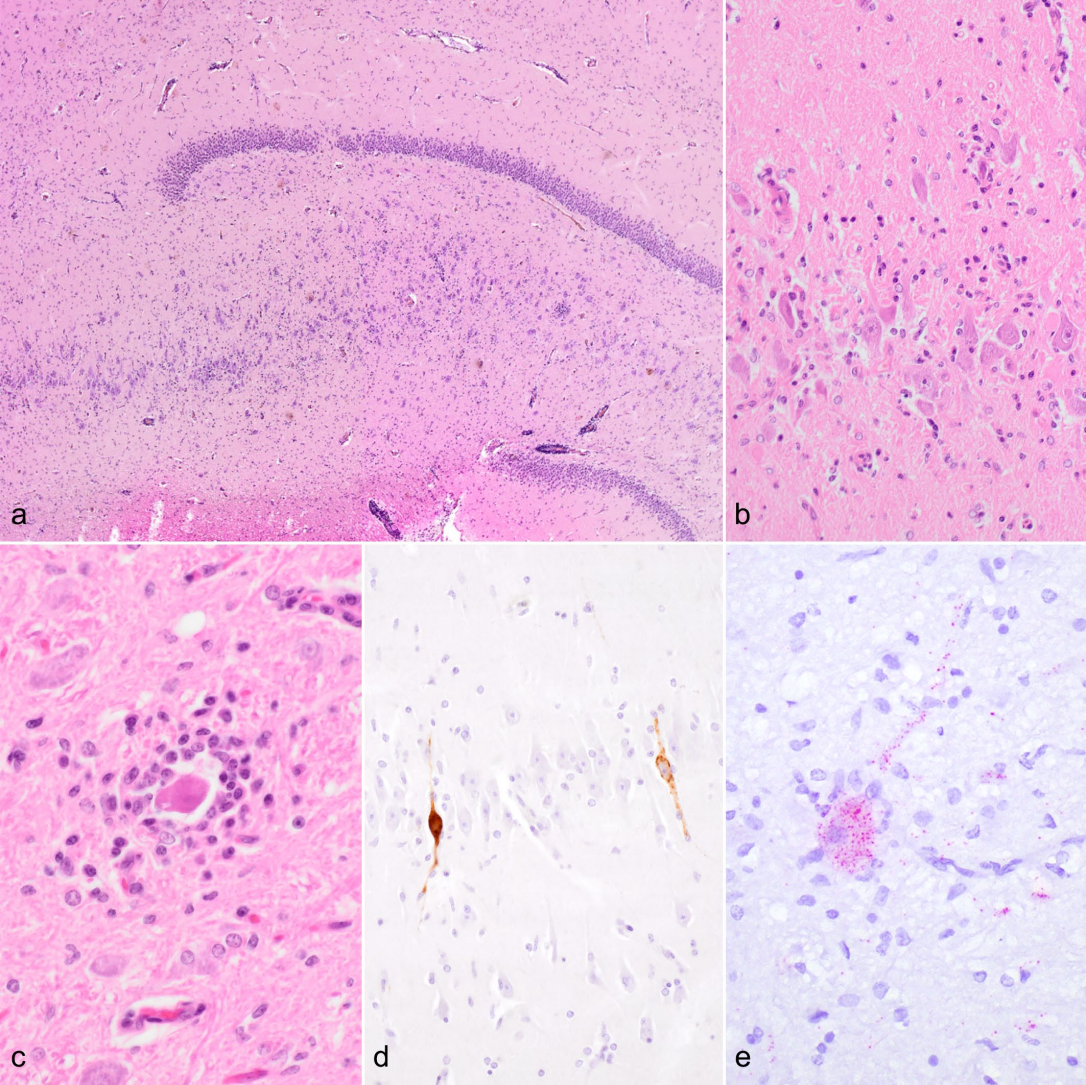
Histopathologic features, immunohistochemistry, and RNAscope in situ hybridization of tick-borne encephalitis virus (TBEV) infections in alpacas. Brain, alpaca. (a) Overview demonstrating neuroparenchymal inflammation in the hippocampus region. Hematoxylin and eosin (HE). (b) Higher magnification of (a) showing severe lymphoplasmacytic and histiocytic inflammation together with microgliosis. HE. (c) A degenerate neuron surrounded by lymphocytes, plasma cells, and histiocytes (neuronophagic nodule) in the medulla oblongata. HE. (d) Intraneuronal immunolabeling for TBEV in the hippocampus region. TBEV immunohistochemistry. (e) Red positive hybridization signals are in neuronal cell bodies and processes in the brain stem. TBEV RNAscope in situ hybridization.

In spring of 2022, an 8-month-old female Huacaya alpaca started with colic-like signs, bruxism, and ventral recumbency. Despite symptomatic therapy including anti-inflammatory medication with meloxicam (Melovem) and dexamethasone (Dexa Ject); antibiotics with cefquinome (Ceffect, Cobactam); vitamin B complex; and physiologic saline containing amino acids, vitamins, and electrolytes (Duphalyte), the animal showed a progressive deterioration, leaving it barely able to raise the head. The alpaca was euthanized and referred to the Vetmeduni Vienna for necropsy, which grossly revealed a body condition score of 2 out of 5; mild hydropericardium; mild, multifocal mucosal erosions in the third gastric compartment; and congestion of parenchymal organs. Routine tissue samples, including the CNS, were collected, fixed in 10% neutral-buffered formalin, embedded in paraffin, sectioned, and stained with hematoxylin and eosin. Selected tissue samples including the brain and spinal cord were first stored at −18°C and the long-term stored at −80°C. Histologic examination of the intestine revealed severe intestinal coccidiosis, which was not further specified. The brain and spinal cord contained a lymphohistiocytic encephalomyelitis, which was predominantly localized to the gray matter of the cornu ammonis region (Fig. 1a, b), as well as in the pons, medulla oblongata, and spinal cord. Associated histologic lesions included perivascular cuffing, neuronal degeneration, neuronophagia (Fig. 1c), capillary dilation, and multifocal glial nodules. In addition, there was a moderate, multifocal lymphohistiocytic leptomeningitis. Formalin-fixed paraffin-embedded sections of the cerebrum at the level of the hippocampus and spinal cord were subjected to immunohistochemistry for TBEV, glial fibrillary acidic protein (GFAP), and ionized calcium-binding adapter molecule 1 (IBA1) detection using an automated immunostainer (Lab Vision AS 360, Lab Vision, Thermo Fisher Scientific, Fremont, California). Antigen retrieval was conducted by pronase for TBEV, whereas no pretreatment was necessary for GFAP and IBA1. Primary rabbit polyclonal Western-TBEV antibody (dilution 1:500, kindly provided by the Department of Virology, Medical University of Vienna); rabbit polyclonal GFAP antibody (Z0334, dilution 1:10000, Dako, Agilent, Santa Clara, California); rabbit monoclonal IBA1 antibody (019-19741, dilution 1:1000, Wako Chemicals, Richmond, Virginia); and horseradish peroxidase polymer (Lab Vision, Thermo Fisher Scientific) were applied, and the reaction was visualized with diaminobenzidine (Lab Vision, Thermo Fisher Scientific). Internal positive controls were used for all markers and isotype controls were also included. Intralesional TBEV immunolabeling was demonstrated in numerous neurons in the brain (Fig. 1d) and spinal cord. GFAP and IBA1 immunolabeling confirmed intense glial cell proliferation of affected regions.

For visualizing viral RNA in the CNS, in situ hybridization was performed on selected brain regions, including the brain stem, cerebellum, and forebrain using RNAscope technology with the RNAscope R (Red) 2.5 detection kit and a TBEV detection probe (Category No. 575601; Advanced Cell Diagnostics, Bio-Techne, Minneapolis, Minnesota) as described previously.^6^ A polymerase chain reaction (PCR) confirmed a horse brain infected by TBEV served as a positive control. Neuronal clusters of all regions of the alpaca brain showed hybridization signal throughout neuronal cell bodies and processes, with high intensity in single pyramidal cells of hippocampal cornu ammonis. These cells also often showed extensive mixed microglial/lymphocytic satellitosis and neuronophagia.

From the frozen samples, a total of 0.1 g of tissue was homogenized in 1 ml phosphate-buffered saline with a TissueLyser II (Qiagen, Hilden, Germany) at 30 Hz for 3 minutes. Nucleic acid was extracted using a TANBead Nucleic Acid Extraction Kit (OptiPure Viral Auto Tube, #M665S46; Taiwan Advanced Nanotech) for magnetic bead–based extraction at an automated TANBead Malstroem 4800 (Taiwan Advanced Nanotech) nucleic acid extraction system according to the manufacturer’s instructions. TBEV-specific nucleic acids were detected by a duplex real-time quantitative reverse transcription-PCR (RT-qPCR) assay developed by Schwaiger and Cassinotti.^21^ that amplifies a 66-bp region in the 3’UTR region of TBEV together with the detection of beta-actin as an internal control according to Toussaint et al.^22^ The RT-qPCR was conducted with a Luna Probe One-Step RT-qPCR Kit (#E3007E, New England Biolabs, Germany) using an annealing temperature of 60°C following the manufacturer’s instructions and included negative and positive controls. The latter one was obtained from a horse with confirmed TBEV infection. The RT-qPCR revealed a TBEV-RNA-specific quantitative cycle value (Cq) of 23.6 and a Cq of 18.6 for the internal control. The positive control showed a TBEV-RNA-specific Cq value of 26.8 and Cq 35 for the internal control.

**Figure 2.**
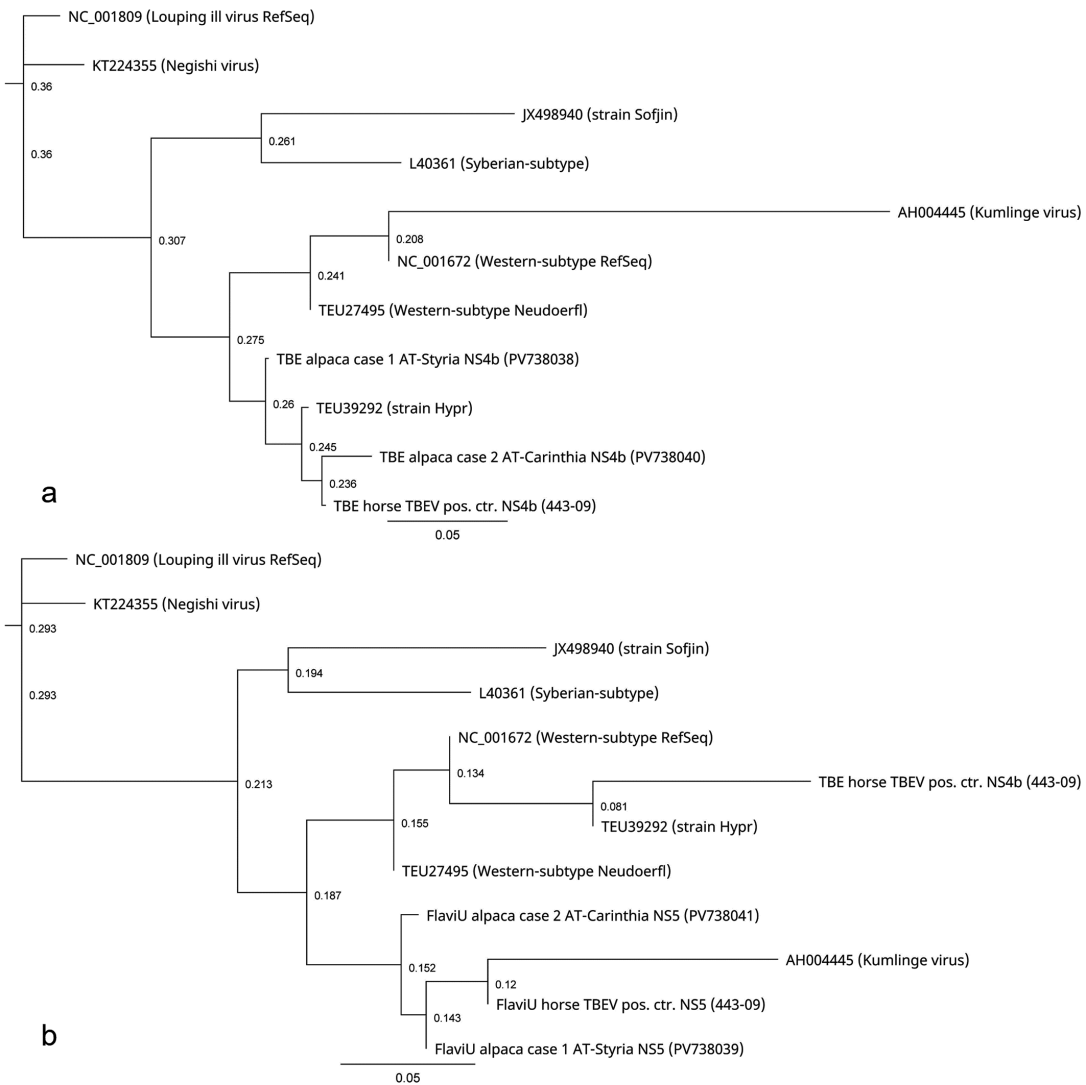
Phylogenetic trees for the tick-borne encephalitis virus (TBEV) (a) and universal pan-flavivirus-specific (FlaviU) (b) sequences of the alpaca cases 1 and 2, the positive control, and their relationship to reference strains of TBEV subtypes and strains belonging to *Orthoflavivirus encephalitidis* according to the NCBI Taxonomy Browser (accessed 18 February 2025). Louping ill virus was included as an outgroup in the tree-building model. Trees were generated using Tamura-Nei as the genetic distance model and Neighbor-Joining as the tree-building method. Scale bars = distances (nucleotide substitution rates). Created with Geneious Prime Version 2025.0.3.

For sequence analysis, 2 different RT-PCRs were conducted for amplification of partial TBEV-specific non-structural protein NS4b (234 bp) and pan-flavivirus-specific NS5 fragments (260 bp) according to Bagó et al^1^ and Patel et al,^15^ respectively, via blast-nucleotide comparison (NCBI, Bethesda, Maryland) and showed > 99% identities to other published TBEV strains deposited in the NCBI GenBank database for both samples (Supplemental Table S1). The TBEV sequence was most closely related (99.21%) to TBEV strains from Switzerland and the Czech Republic, and the pan-flavivirus sequences (98.57%) to TBEV strains from Slovenia, Germany, Italy, Switzerland, and Sweden. The positive control showed the closest relationships to hyperstrain variants of TBEV for both sequences (100.0% and 99.2%). The nucleotide identity between case 1 and the positive control samples was 98.53% (1/67) and 98.31% (4/233) for the TBEV- and pan-flavivirus-specific sequences, respectively (Supplemental Figure S1). Phylogenetic trees for the TBEV (Fig. 2a) and pan-flavivirus-specific sequences (Fig. 2b) were generated using Tamura-Nei as the genetic distance model and Neighbor-Joining as the tree-building method and created with Geneious Prime (Version 2025.0.3). To investigate the relationship between subtypes and strains of TBEV and the sequenced samples, reference strains of *Orthoflavivirus encephalitidis* according to the NCBI Taxonomy Browser (accessed 18 February 2025) and, as outgroup louping ill virus, were included in the tree-building model. Both trees confirmed the closest relationship to the Western subtype of TBEV (Fig. 2a, b).

## Case 2

In early summer of 2022 an 8-year-old, male-neutered Huacaya alpaca showed weakness, reduced feed intake, and muscle trembling, followed by progressive neurologic signs including seizures, paresis, and blindness. The veterinary practitioner suspected listeriosis, and antibiotic and symptomatic therapy was administered. Prior to this, the animal had been treated against parasites with doramectin (Dectomax). Due to progressive worsening of the clinical status, the animal was euthanized 3 days later and submitted for postmortem examination at the Carinthian Institute for Food Safety and Veterinary Medicine. The alpaca had a body condition score of 3 out of 5, was slightly anemic and jaundiced. Histologic examination of the CNS revealed a moderate lymphohistiocytic meningoencephalomyelitis predominantly in the gray matter, comparable to that in case 1, that was primarily localized to the brainstem and cervical spinal cord. Additional findings included mild hydropericardium, moderate macrovesicular steatosis, multifocal petechiae in the urinary bladder mucosa, and moderate hemorrhagic-necrotizing jejunitis with numerous intralesional bacteria. Aerobic and anaerobic bacteriological cultures of jejunum were negative. Fecal flotation revealed a mild infestation with *Eimeria* species that was not further specified. Immunohistochemistry of affected brain regions for TBEV, as previously described, was negative. RNAscope in situ hybridization for TBEV RNA, as previously described, showed a mild to marked hybridization signals in multiple individual neuronal cell bodies and processes throughout the mesodiencephalic region (Fig. 1e). Only a few neuronal processes showed similar labeling intensities as the hippocampal neurons in case 1. Also in this animal, positive cells were frequently associated with gliosis, microglial-lymphocytic satellitosis, and occasional early neuronophagia. To exclude notifiable infectious diseases, tissue samples from the brain and cervical spinal cord were tested, using direct fluorescent antibody testing for rabies, as previously described,^28^ and specific RT-qPCRs for West Nile virus lineages 1 and 2,^11^ borna disease virus-1,^20^ Eastern equine encephalitis virus, Western equine encephalitis virus, and Venezuelan equine encephalitis virus, according to the European Union reference laboratories.^12,24^ In addition, a universal pan-flavivirus RT-PCR^19^ was performed. The direct fluorescent antibody test for rabies virus and specific RT-qPCRs for West Nile virus-1/2, borna disease virus-1, Eastern equine encephalitis virus, Western equine encephalitis virus, and Venezuelan equine encephalitis virus were negative. The pan-flavivirus heminested RT-PCR targeting the *NS5* showed specific PCR bands (214 bp) from the brain.^19^ Therefore, RNA was extracted, and PCR and sequence analyses were conducted as previously described. The RT-qPCR revealed a TBEV-RNA-specific Cq of 26.3 and a Cq of 19.4 for the internal control. The TBEV sequence was most closely related (98.48%) to TBEV strains from Italy, Finland, Belgium, and Germany, and the pan-flavivirus sequences to TBEV strains (98.07%) from Slovakia (Supplemental Table S1). Sequence alignments and phylogenetic analyses confirmed a similar close relationship of sequences to the Western subtype of TBEV as found for case 1 (Supplemental Figure S1 and Fig. 2a, b). The nucleotide identity between both cases and between case 2 and the positive control samples was 95.93% (5/118) and 97.86% (3/137) for the TBEV-specific sequences, respectively, and 97.92% (5/235) and 98.01% (5/246) for the pan-flavivirus-specific sequences, respectively. Phylogenetic trees were generated and created as previously described. The sequence information is available in GenBank (accession numbers: PV738038 to PV738041) and in the Supplemental Materials.

The animals in this study came from Styria (case 1) and Carinthia (case 2), which are TBEV-endemic federal states in the southeastern part of Austria. Correlating with the seasonal activity of ticks, disease outbreak emerged in the spring and early summer. Due to the conventional kind of extensive husbandry on pastures, alpacas are at risk to be infested by ticks.^3^ Prevention strategies, such as regular animal inspections and reduction of potential sources of exposure, are limited due to physiologic conditions (eg, tight fur) and their preference of keeping distant from humans. In addition, until now, vaccination or antiviral therapy for TBE is not available in veterinary medicine.

The alpacas presented with neurologic signs, including muscle weakness progressing to paresis, convulsions, seizures, and blindness, consistent with those seen in dogs with TBE.^18^

Reports of histopathologic findings in natural TBEV infections in domestic animals are generally scarce. In dogs, these include moderate to severe, multifocal lymphoplasmacytic and histiocytic meningoencephalomyelitis that affects gray and white matter, neuronal necrosis, neuronophagia, perivascular cuffing, gliosis, variable demyelination, and white matter edema.^18,23,26^ Lesions are predominantly distributed within the cerebral cortex, hippocampus, basal ganglia, brainstem, cerebellum, and sometimes the spinal cord.^10^ Histopathologic findings in the CNS and their distribution in the presented alpacas are quite similar but were primarily in the brainstem and cervical spinal cord of case 2.

Case 2 demonstrated a lack of immunolabeling for TBEV. In some reports of natural TBE infections in dogs, sheep, and horses, which were otherwise confirmed by molecular techniques, immunohistochemical examination failed to detect viral antigen.^2,6,26^ Subsequently, RNAscope technology was applied and successfully demonstrated hybridization signals. We conclude that immunohistochemistry may sometimes be insufficient for TBEV detection. The sequencing and phylogenetic results indicate that the TBEV strains of the 2 alpaca cases are closely related but originate from different regions and are reassortants of different European strains of the Western subtype of TBEV.

In recent years, South American camelids, including alpacas, llamas, and guanacos, have gained popularity, and are now facing previously unknown pathogens outside their natural habitat. In general, the current knowledge of viral diseases, including the incidence, susceptibility, and clinical picture in these species is rudimentary or even unknown, and only a few of them have been identified so far.^9^ Therefore, emerging diseases such as TBE should be considered in South American camelids.

## Supplemental Material

sj-pdf-1-vet-10.1177_03009858251362432 – Supplemental material for Natural tick-borne encephalitis in 2 Huacaya alpacas (Vicugna pacos) Supplemental material, sj-pdf-1-vet-10.1177_03009858251362432 for Natural tick-borne encephalitis in 2 Huacaya alpacas (Vicugna pacos) by Denise Thaller, Claudia Schulz, Angelika Auer, Zoltán Bagó, Sandra Revilla-Fernández, Michael D. Mansfeld, Kaspar Matiasek and Andrea Klang in Veterinary Pathology
