# Supplementary material for: Natural tick-borne encephalitis in 2 Huacaya alpacas (Vicugna pacos)
Source: Vet Pathol. 2025 Aug 19;63(1):116–21. doi: 10.1177/03009858251362432 (PMC12743117; doi:10.1177/03009858251362432)
Supplement: sj-pdf-1-vet-10.1177_03009858251362432 – Supplemental material for Natural tick-borne encephalitis in 2 Huacaya alpacas (Vicugna pacos) [file sj-pdf-1-vet-10.1177_03009858251362432.pdf]

Supplemental Materials

Natural tick-borne encephalitis in two Huacaya alpacas (*Vicugna pacos*)

Denise Thaller, Angelika Auer, Claudia Schulz, Zoltán Bagó, Sandra Revilla-Fernández, Michael Dieter Mansfeld, Kaspar Matiasek, and Andrea Klang

Tick-borne encephalitis virus (TBEV) and pan-flavivirus nucleotide sequences of samples from alpaca cases 1 and 2 and the positive control (pos. ctr.) obtained by amplification and sequencing of partial TEBV-specific none-structural protein (NS) *NS4b* (234 bp) and pan-flavivirus-specific *NS5* fragments (260 bp) (FlaviU) according to Bagó et al.[1] and Patel et al.,[14] respectively.

Alpaca case 1

>TBE alpaca case 1 AT-Styria NS4b (PV738038)
CCATGGAGTGAATGGACGAATGTGGACATCCAGCCAGCGAGGTCCTGGGGAACCTATGTGCTGG
TGGTGTCTCTGTTTACACCTTACATCATCCACCAACTGCAGACCAAAATACAACAACTTGT

>FlaviU alpaca case 1 AT-Styria NS5 (PV738039)
GGGAAGAGAGAGAAGAAACTGGGAGAGTTCGGAGTGGCGAAAGGAAGTCGGGCCATTTGGTAC
ATGTGGCTGGGGAGTCGCTTTCTGGAGTTCGAGGCTCTTGGATTCTTGAATGAGGACCATTGGG
CCTCTAGAGAGTCCAGTGGAGCTGGAGTCGAGGGAATAAGCTTGAACTACCTGGGCTGGCACCT
CAAGAAGTTGTCGACCCTGAATGGAGGACTCTTCTATGCAGATGACACC

Alpaca case 2

>TBE alpaca case 2 AT-Carinthia NS4b (PV738040)
AATGAGATGGGCTTTCTGGAGAAGACCAAGGCAGACTTGTCCACGGTGCTGTGGAGTGAACAGG
AGGAACCCCGGCCATGGAGTGAATGGACGAATGTGGACATCCAGCCAGCGAGGTCTTGGGGGA
CCTATGTGCTGGTGGTGTCTCTGTTTACACCTTACATCATCCACCAGCTGCAGACCAAAATCCAA
CAACTC

>FlaviU alpaca case 2 AT-Carinthia NS5 (PV738041)
ATGGGGAAGAGAGAGAAGAAACTGGGAGAGTTTGGAGTGGCGAAAGGAAGTCGGGCCATTTGG
TACATGTGGCTGGGGAGTCGCTTTCTGGAGTTCGAGGCTCTTGGATTCTTGAATGAAGACCATTG
GGCCTCTAGAAAGTCCAGTGGAGCTGGAGTTGAGGGAATAAGCTTGAACTACCTGGGCTGGCAC
CTCAAGAAGTTGTCGGCCCTGAATGGAGGACTCTTCTATGCAGATGACACCGCGGGCTGGGACA
C

Positive control

>TBE horse TBEV pos. ctr. NS4b (443-09)
GAGATGGGTTTTCTGGAGAAGACCAAGGCAGACTTGTCCACGGTGCTGTGGAGTGAACGGGAG
GAACCCCGGCCATGGAGTGAATGGACGAATGTGGACATCCAGCCAGCGAGGTCCTGGGGGACC
TATGTGCTGGTGGT

>FlaviU horse TBEV pos. ctr. NS5 (443-09)
AAGAGAGAGAAGAAACTGGGAGAGTTCGGAGTGGCGAAGGGAAGTCGGGCCATTTGGTACATGT
GGCTGGGGAGTCGCTTTCTGGAGTTCGAGGCTCTTGGATTCTTGAATGAAGACCATTGGGCCTC
TAGAGAGTCCAGTGGAGCTGGAGTTGAGGGAATAAGCTTGAACTACCTGGGCTGGCACCTCAAG
AAGTTGTCAACCCTGAATGGAGGACTCTTCTATGCAGATGACACCGCGGGCTGGGACAC

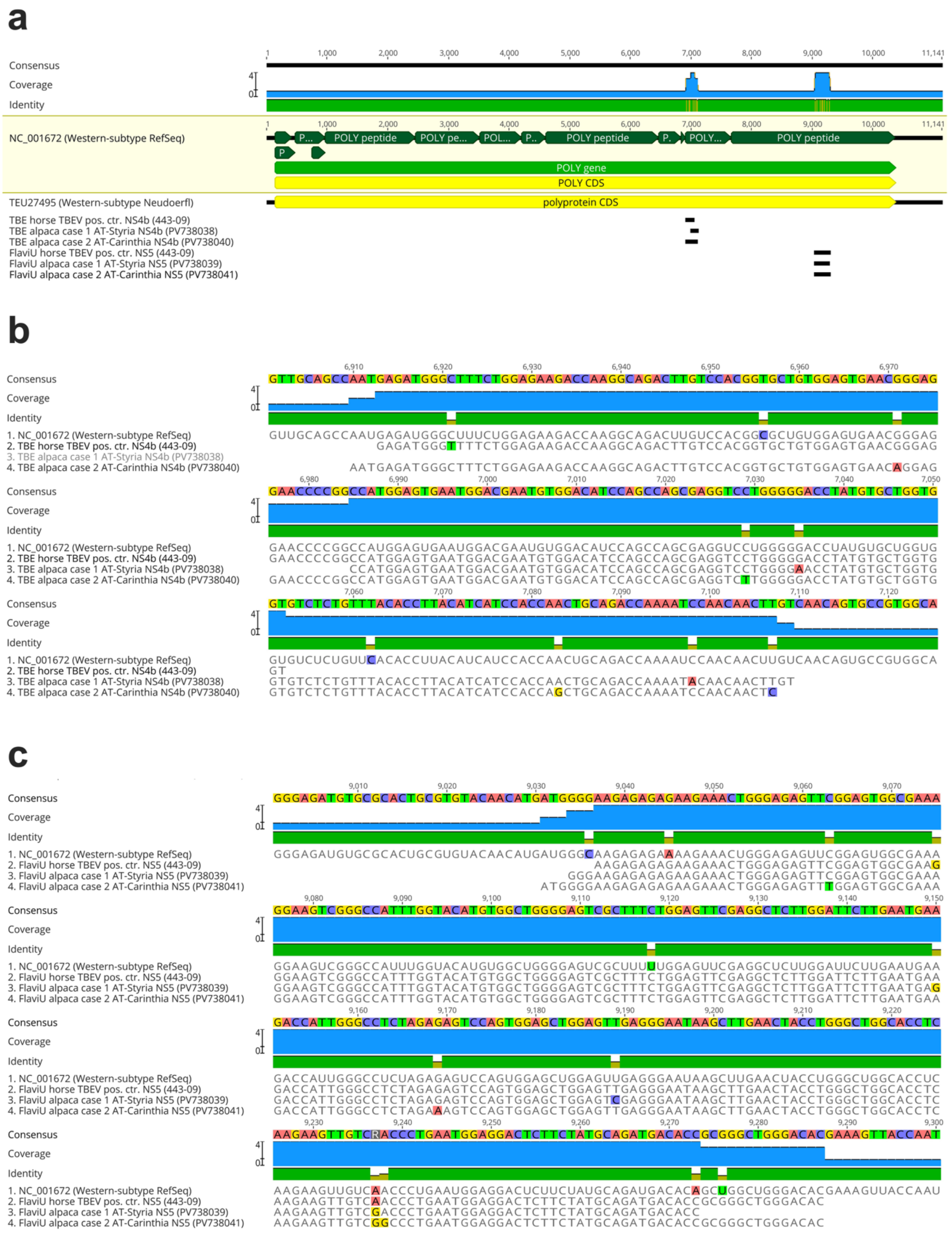

Supplemental Figure S1. (a-c) Alignment of tick-borne encephalitis virus (TBEV) and universal pan-flavivirus-specific (FlaviU) nucleotide (nt) sequences obtained from two alpaca brain samples (cases 1 and 2) and the positive control (pos. ctr.). **(a)** Assembly of sample sequences to reference sequences (RefSeq, Neudoerfl) of the TBEV Western subtype in the region of the non-structural protein (NS) genes *NS4b* and *NS5*, respectively. **(b)** Alignment of TBEV-specific sample sequences and visualization of nucleotide exchanges in the region *NS4b*. **(c)** Alignment of FlaviU-specific sample sequences and visualization of nucleotide exchanges in the region *NS5*. Created with Geneious Prime Version 2025.0.3. (GenBank Accession numbers PV738038 to PV738041)

Supplemental Table S1. Results of blast-nucleotide sequence comparison searches using NCBI GenBank for tick-borne encephalitis virus (TBEV) strains and/or geo locations related to the TBEV-RNA-positive alpaca samples and the positive control.

Sample	RT-PCR assay	Gen region (RefSeq NC_001672)	%identity to sample	Accession#	Strain and/or geo location of European subtype
TBE horse TBEV positive control	TBE[1]	NS4b	100.00	KP716975.1<br>MT228626.1<br>KP716977.1<br>KP716974.1<br>MT228627.1<br>OP037819.1<br>MT228625.1<br>KP716976.1<br>MT228628.1	Hyper strain variants
TBE alpaca case 1 Styria	TBE[1]	NS4b	99.20	KJ922513.1<br>OQ555317.1<br>KJ922512.1	Petracova (Czech Republic)<br>GR_UM/Switzerland/2022<br>Kubinova (Czech Republic)
TBE alpaca case 2 Carinthia	TBE[1]	NS4b	98.48	PP782056.1<br><br>MK801804.1<br>PQ470587.1<br>MK801807.1<br>MK801808.1<br>MK801805.1<br>KX268728.1	Chamois/Italy-BG/143872/2023_Lombardy<br>Sipoo-4-Finland-2013<br>Wanze01 (Belgium)<br>Sipoo-22-Finland-2013<br>Sipoo-23-Finland-2013<br>Sipoo-8-Finland-2013<br>MucAr-HB-171/11 (Germany)
FlaviU horse positive control	FlaviU[2]	NS5 (RNA-dependent RNA polymerase)	99.20	U39292.1<br>MT228626.1<br>MT228627.1<br>OP037819.1<br>MT228625.1<br>MT228628.1<br>KP716978.1	Hyper strain variants
FlaviU alpaca case 1	FlaviU[2]	NS5 (RNA-dependent RNA polymerase)	98.57	OQ889248.1<br>GQ266392.1<br>OM084948.1<br>OQ555316.1<br><br>OQ555317.1<br><br>OQ889230.1<br>MT581212.1	2022-P116-S1 (Slovenia)<br>AS33 (Germany: Amberg)<br>TBEV-Eu (Italy)<br>SG_RM/Switzerland/2022 (Switzerland: St. Gallen)<br>GR_UM/Switzerland/2022 (Switzerland: Grison)<br>2018-P45-S1 (Slovenia)<br>93/783 (Sweden)
FlaviU alpaca case 2	FlaviU[2]	NS5 (RNA-dependent RNA polymerase)	98.05	KC835597.1<br><br>KC835595.1<br>KC835596.1	CGI223 (Slovakia: Zahorska Ves)<br>114 (Slovakia: Plastovce)<br>285 (Slovakia: Malacky)

[1] TBE: TBEV-RNA-specific RT-PCR according to Bagó et al.[1] (Primer TBE1: 5´-GGACTGGTTGCAGCCAATGA-3´; (TBE4): 5´-AGATGCCACGGCACTGTTGA-3´),

[2] FlaviU: universal pan-flavivirus-specific RT-PCR according to Patel et al.[14] (Primer S-F: 5´-TACAACATGATGGGGAARAGAGARAA-3´; AS2-R: 5´-GTGTCCCAGCCNGCKGTGTCATCWGC-3´)
